# Cannabinoid Hyperemesis Encounters After Medical Legalization in Oklahoma

**DOI:** 10.7759/cureus.46465

**Published:** 2023-10-04

**Authors:** Randal Riha, Ryan Winchell, Danielle Safo, Joshua Gentges

**Affiliations:** 1 Department of Emergency Medicine, St. John's Ascension, Tulsa, USA; 2 Department of Emergency Medicine, University of Oklahoma Health Sciences Center, Tulsa, USA

**Keywords:** legalization, medical marijuana, cannabis, public policy, public health, toxicology, chs

## Abstract

Introduction

Medical cannabis has recently become legal in Oklahoma. Cannabinoid hyperemesis syndrome (CHS) is severe nausea, vomiting, and often abdominal pain typically seen in heavy users of cannabis. The aim of this study is to determine whether emergency department (ED) diagnoses of CHS have increased after medical legalization.

Methods

We performed a retrospective chart review study of equivalent time periods prior to and after the first legal sales of medical cannabis in Oklahoma. Data were gathered from a single urban ED of adult patients with diagnosed or suspected CHS. We analyzed data using a chi-square analysis of CHS cases as a proportion of total ED visits.

Results

Diagnosed and suspected CHS visits increased from 43 cases in the eight months preceding the first legal sale to 62 cases in the eight months after legalization. This represents a statistically significant increase in ED visits for CHS (p = 0.026). Total ED encounters were 30,437 and 28,362, respectively, during those time periods. The proportion of visits for CHS was much higher (220/100000 vs 13.3/100000) than previously reported in the literature. The pre-legalization and post-legalization groups did not differ by age, sex, history of GI illness and diabetes, pregnancy, or other drug use.

Conclusion

We observed a statistically significant increase in ED visits for CHS after the first legal sales of medical cannabis in Oklahoma. Our high proportion of ED visits for CHS could be related to study design, increased provider awareness, high THC levels in Oklahoma’s medical cannabis, or increased numbers of cannabis users after legalization. Increases in ED visits for CHS and other cannabinoid-related illnesses must be weighed against the positive effects for cannabis users by policymakers.

## Introduction

*Cannabis sativa* (cannabis) has been used by humans for millennia, whether for medicinal use, religious use, or for its mind and/or mood-altering properties. There are many claimed medicinal properties of cannabis, but to this date, the United States Food and Drug Administration (FDA) has granted approval only for specific cannabis derivatives to treat seizures associated with rare forms of epilepsy, appetite, and weight loss in HIV/AIDS, as well as nausea and vomiting associated with chemotherapy [1]. Currently, marijuana is listed as a Schedule 1 substance by the DEA, which states that marijuana has a high potential for abuse and no currently accepted medical use [2]. Schedule 1 classification mandates specific research protocols and safety requirements, which makes medicinal marijuana research potentially burdensome.

Though cannabis is thought to have antiemetic properties, its use can paradoxically cause severe nausea and vomiting. Cannabinoid hyperemesis syndrome (CHS) is the syndrome of severe nausea and vomiting and is often accompanied by abdominal pain, typically in chronic users of cannabis or its derivative products [3]. Most patients with CHS are daily cannabis consumers, and occasional consumers (weekly or less) seldom develop CHS [4]. The mechanism is unknown but may be related to the overstimulation of cannabinoid receptors or the buildup of toxic metabolites in fatty tissue [4]. CHS often occurs in a cyclical nature and is frequently reported to be relieved by warm showers or baths. The vomiting in CHS is often quite severe and forceful, being colloquially termed “scromiting,” a combination of the words “scream” and “vomiting” first described in the medical literature in 2004 [5].

Vomiting associated with CHS can have severe consequences. Forceful vomiting can result in esophageal injury, including Mallory-Weiss tear, or Boerhaave syndrome [6]. Unabated vomiting can result in dehydration, electrolyte derangement, acid-base disorder, and kidney injury and can potentially cause any number of unforeseen complications in patients with chronic disease [5]. CHS often requires medical treatment and even hospital admission for electrolyte and fluid repletion as well as symptomatic control [6]. Treatment is symptomatic, but the most effective way to resolve CHS is the cessation of cannabis use [5].

Laws allowing for increased legal access to cannabis have rapidly propagated throughout the United States, starting with California Proposition 215, passed in 1996, which granted medical access to cannabis [7], and more recently with a trend toward legalized access to recreational cannabis. As of February 2023, 37 states have a legal framework for medical cannabis, and there are 21 states that have enacted regulations for non-medicinal use of cannabis [8]. On June 26, 2018, Oklahoma legalized the medical use of cannabis, with the first sales occurring in November of 2018. Previous studies have shown an increase in CHS after the liberalization of medical or recreational cannabis laws [9-11]. Wang et al. [9] found a small (IRR, 1.03; 95% CI, 1.01-1.05) increase in all vomiting complaints in Colorado EDs after cannabis legalization. Yeung et al. [10] found a 45% increase in cannabis-related visits after legalization in Alberta. Bollom et al. [11] found a significant increase in cannabis use disorder combined with EDD visits for vomiting between 2006 and 2013, during which cannabis was becoming legal for medical use in many states. The aim of this study is to determine if there has been an increase in emergency department (ED) diagnoses of CHS after medical sales began in Oklahoma.

## Materials and methods

Study design

We performed a retrospective observational study of ED visits for CHS prior to and after the legalization of medical cannabis in Oklahoma. Records of ED encounters were reviewed to identify visits with diagnosed or likely CHS based on physician note. The time period of the question was an eight-month period prior to and after the first legal cannabis sales in Oklahoma. The first legal cannabis sales were in November of 2018, and thus, the two arms were from March 1, 2018, to October 31, 2018, for pre-legalization and November 1, 2018, to June 30, 2019, for post-legalization. This time frame of eight months prior to legal cannabis sales was chosen because a new electronic medical record system was established at the site on March 1, 2018, and we wanted to minimize bias by including data from an entirely different source.

Inclusion criteria

The patient population of this study was patients of this ED in the above time frame that were evaluated by a physician or midlevel provider. Likely, CHS was determined if the patient was diagnosed with nausea and vomiting, with no identified or suspected causative etiology listed in the chart, and had a history of greater than weekly cannabis use. The primary outcome measured was the total number of visits in the time period prior to and after the legalization of medical cannabis, as well as establishing any trend or accelerating trend in increasing the number of visits for CHS. Patients categorized as having CHS were those with an International Classification of Disease 10 (ICD10) code assigned during that ED visit for CHS, or any of the ICD codes for vomiting or cyclical vomiting without expressed causative etiology and chart review, suggesting CHS as a diagnosis. For patients in the latter category, their chart was reviewed, including reading the applicable physician or midlevel provider note and reviewing their social history. Patients were defined as CHS if they reported cannabis use greater than weekly, cyclic, severe nausea and vomiting without diarrhea, and no other etiology was present in the chart.

Exclusion criteria

Patients that had a disposition of “dead on arrival” (DOA), “left without being seen” (LWBS) by a medical provider, or sent to “labor & delivery” (L&D) were excluded from the study, as no physician or midlevel provider note would be present in the chart. If the note identified a cause or suspected cause of the nausea and vomiting, they were excluded from the CHS group. If their social history did not include cannabis use greater than weekly, they were also excluded from that group. Patients with ICD10 codes, including diarrhea, were excluded from the cannabinoid hyperemesis group, as the diarrheal illness is not typical of CHS. Minors under 18 years old were excluded from the group, as there is additional regulation pertaining to legal access to cannabis for minor patients. 

Statistical analysis

We compared the proportion of ED patients with CHS prior to legalization to the proportion after legalization, using a chi-square test for differences in population incidence. In addition, we gathered CHS patient age and sex demographic data, other drug or alcohol use, history of diabetes, GI disorders, and pregnancy status to compare demographics and risk factors for the pre- and post-legalization groups. These data were compared using the Kruskal-Wallis rank-sum test.

## Results

**Figure 1:**
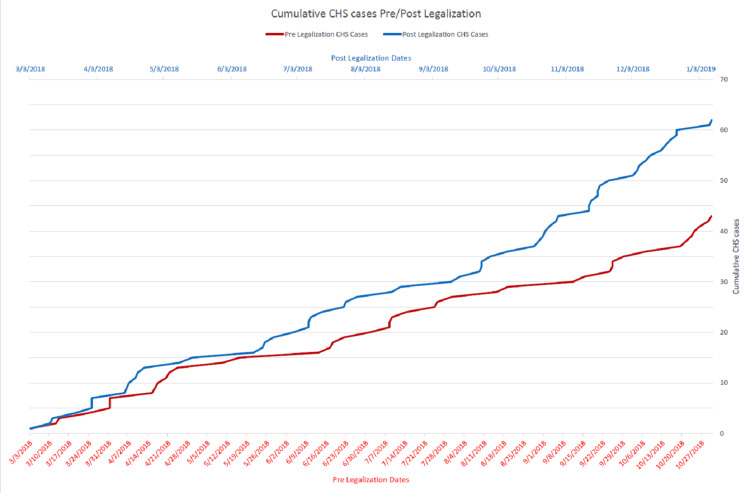
Cumulative CHS cases before and after cannabis legalization in Oklahoma CHS, cannabinoid hyperemesis syndrome

From the pre-legalization period of March 1, 2018, to October 31, 2018, there were 30,437 visits to this ED, excluding patients DOA, LWBS, or transferred to L&D. Of these, 43 were diagnosed with CHS or suspected of CHS, making up 0.14% of visits. From the time of the first legal sales on November 1, 2018, to June 30, 2019, there were 28,362 visits to this ED, excluding patients DOA, LWBS, or sent to L&D. Of these, 62 were diagnosed with CHS or suspected of CHS, making up 0.22% of visits. Chi-square analysis yielded a chi-square statistic of 4.92. This represented a statistically significant increase in the proportion of ED visits (p = 0.026). Cumulative totals of CHS patients in both the pre- and post-legalization periods are shown in Figure 1.

We performed a secondary analysis using logistic regression and Kruskal-Willis testing to analyze whether the pre- and post-legalization CHS patients were equivalent (Table 1). There were no statistically significant differences between the pre- and post-legalization groups by age, sex, alcohol use, other drug use, frequency of cannabis use, diabetes, history of GI disorder, or pregnancy. In both groups, over 70% of patients used cannabis daily. Of 12 unique diabetic patients in the study, seven (58%) had a known history of gastroparesis. There were also no significant interactions between any descriptive variables.

**Table 1:** Characteristics of pre- and post-legalization CHS patients CHS, cannabinoid hyperemesis syndrome

		Age	Sex (M)	Alcohol use	Other drug use	Daily cannabis use	Diabetes	GI disorder	Pregnancy
Pre-legalization		33.9	0.42	0.42	0.11	0.71	0.18	0.34	0.016
Post-legalization		32.9	0.49	0.53	0.12	0.72	0.047	0.21	0
p-value		0.5	0.48	0.067	0.11	0.3	0.073	0.19	0.14

## Discussion

We have identified a statistically significant increase in ED visits for diagnosis of CHS after the legalization of medical cannabis in Oklahoma when compared to an equivalent time period prior to legalization. These results are not unexpected and in line with prior studies showing an increase in visits after liberalization of cannabis laws [9-11]. Our results show a much higher proportion of ED visits for CHS (220/100000 vs 13.3/100000) than previously reported [11], which we believe is due to multiple causes. There may be an increase in practitioner knowledge of CHS since the previous study, leading to higher rates of diagnosis. Because our study showed a higher proportion of ED visits for CHS, a true increase in CHS cases is probable. It is also possible that our intensive methodology to identify CHS cases that were not ICD-10 coded as such by in-depth chart review led to our higher proportion of CHS visits. Conversely, the strict exclusion criteria may have underreported CHS visits. Follow-up research on the long-term trends of CHS visits to the ED would be necessary to determine whether increases in a post-legalization framework continue to rise or, more likely, a plateau at some steady state value. We considered an additional study arm to include more recent ED visits, but this was determined to have been complicated by the COVID-19 pandemic and associated changes in ED visits, as well as COVID-19 visits for nausea and vomiting.

This study was at a single site, and results may not be extrapolatable to other sites, especially in areas where cannabis laws differ from Oklahoma's medical framework. We do not establish a causal connection between CHS and medical marijuana legalization in Oklahoma. One theory is that increased availability results in an increase in number of cannabis users, which has been demonstrated in previous studies [12]. Additionally, the concentration of cannabinoids, specifically THC, has been widely documented to have increased over the last two decades [13]. A legal framework would likely result in the distribution of more potent strains that may be more likely to cause CHS. An increase in visits may also be partially attributable to increased physician and midlevel provider awareness of CHS and specifically asking patients about their cannabis use. Though we attempted to minimize this as a limitation in this study by performing a chart review of patients with no attributable cause of nausea and vomiting for cannabis use, the increased awareness of CHS may result in increased documentation of cannabis use in patients’ medical charts.

There are known benefits to medical cannabis that should be weighed against findings, such as ours, of increased risk of CHS or other cannabis-related side effects. The Committee on the Health Effects of Marijuana from the National Academies of Sciences, Engineering, and Medicine reviewed the literature on the therapeutic effects of cannabis [14]. They found a 64% reduction in the use of opioids in chronic pain and a moderate effect size for pain relief in chronic pain patients. Cannabinoids are effective in treating chemotherapy patients with intractable nausea: a Cochrane review from 2015 [15] showed that cannabinoids were as effective as standard antiemetics and were preferred by patients. They are also effective in relieving spasticity from multiple sclerosis, but the evidence for spinal cord injury is insufficient [14]. Cannabinoids may also improve sleep quality and sleep disturbance [16]. There is limited or no evidence for the treatment of other conditions [16].

One interesting finding was that a large proportion (7/12) of diabetics with CHS had a previous diagnosis of gastroparesis. While this population was small and this study was not designed to specifically evaluate for diabetic gastroparesis, we did note that greater than half of diabetics with this diagnosis were notable because of the possible interaction between CHS and diabetic gastroparesis. It is possible that there is a predisposition of diabetics with gastroparesis to CHS or that emergency practitioners are attributing CHS to cases of gastroparesis. Additionally, diabetics using cannabis may be diagnosed as having gastroparesis when cannabis is the actual underlying cause of their nausea and vomiting. Additional study in this area is warranted.

## Conclusions

The results of this study suggest that liberalization of cannabis laws in Oklahoma was associated with an increase in ED visits for CHS. With a trend toward nationwide liberalization of cannabis laws, both medical and recreational, and specifically a push toward legal recreational cannabis sales in Oklahoma, medical practitioners should expect to see a rise in visits related to complications secondary to cannabis use. We suggest further study of CHS, including studies gathering larger demographic data, frequency of use, and identification of the source of consumed cannabis (homegrown, dispensary purchased, vs black market) such that likely affected cannabis users can be effectively targeted by public health campaigns. Policymakers should weigh the positive effects of cannabinoid use for medical patients against the negative effects, including increased ED visits for cannabis side effects.
